# Barriers to Infection of Human Cells by Feline Leukemia Virus: Insights into Resistance to Zoonosis

**DOI:** 10.1128/JVI.02119-16

**Published:** 2017-02-14

**Authors:** Anne Terry, Anna Kilbey, Asif Naseer, Laura S. Levy, Shamim Ahmad, Ciorsdaidh Watts, Nancy Mackay, Ewan Cameron, Sam Wilson, James C. Neil

**Affiliations:** a MRC University of Glasgow Centre for Virus Research, University of Glasgow, Glasgow, United Kingdom; b School of Veterinary Medicine, University of Glasgow, Glasgow, United Kingdom; c Tulane University, New Orleans, Louisiana, USA; Johns Hopkins University

**Keywords:** feline leukemia virus, restriction factors, APOBEC, zoonosis

## Abstract

The human genome displays a rich fossil record of past gammaretrovirus infections, yet no current epidemic is evident, despite environmental exposure to viruses that infect human cells *in vitro*. Feline leukemia viruses (FeLVs) rank high on this list, but neither domestic nor workplace exposure has been associated with detectable serological responses. Nonspecific inactivation of gammaretroviruses by serum factors appears insufficient to explain these observations. To investigate further, we explored the susceptibilities of primary and established human cell lines to FeLV-B, the most likely zoonotic variant. Fully permissive infection was common in cancer-derived cell lines but was also a feature of nontransformed keratinocytes and lung fibroblasts. Cells of hematopoietic origin were generally less permissive and formed discrete groups on the basis of high or low intracellular protein expression and virion release. Potent repression was observed in primary human blood mononuclear cells and a subset of leukemia cell lines. However, the early steps of reverse transcription and integration appear to be unimpaired in nonpermissive cells. FeLV-B was subject to G→A hypermutation with a predominant APOBEC3G signature in partially permissive cells but was not mutated in permissive cells or in nonpermissive cells that block secondary viral spread. Distinct cellular barriers that protect primary human blood cells are likely to be important in protection against zoonotic infection with FeLV.

**IMPORTANCE** Domestic exposure to gammaretroviruses such as feline leukemia viruses (FeLVs) occurs worldwide, but the basis of human resistance to infection remains incompletely understood. The potential threat is evident from the human genome sequence, which reveals many past epidemics of gammaretrovirus infection, and from recent cross-species jumps of gammaretroviruses from rodents to primates and marsupials. This study examined resistance to infection at the cellular level with the most prevalent human cell-tropic FeLV variant, FeLV-B. We found that blood cells are uniquely resistant to infection with FeLV-B due to the activity of cellular enzymes that mutate the viral genome. A second block, which appears to suppress viral gene expression after the viral genome has integrated into the host cell genome, was identified. Since cells derived from other normal human cell types are fully supportive of FeLV replication, innate resistance of blood cells could be critical in protecting against cross-species infection.

## INTRODUCTION

Completion of the draft human genome sequence in 2001 revealed that a remarkable ∼8% of the genome consists of retrovirus-like elements (1). Although the elements found to date are replication defective, many are related to gammaretroviruses currently circulating as infectious agents in a range of mammalian and avian species (2). Moreover, the ability of gammaretroviruses to cross species boundaries is clear from the fossil record of endogenous viruses and from evidence of recent jumps across wide species barriers, from rodents to primates (gibbon ape leukemia virus [GaLV]) and marsupials (koala retrovirus [KoRV]) (3–5).

The long-standing observation of the ability of some variants of feline leukemia virus (FeLV), a feline gammaretrovirus, to replicate to high titers in human cells *in vitro* led to early concerns with regard to zoonotic spread (6, 7). FeLVs represent important pathogens of the domestic cat that are capable of cross-species spread to endangered feline species (8, 9). Despite widespread domestic contact with FeLV-infected cats, which can shed virus in saliva and other body fluids, a series of careful studies has shown no evidence of serological responses in exposed individuals (10). Human serum factors that lyse or inactivate gammaretroviruses (11, 12) offer a significant obstacle to cross-species spread but appear insufficient to account fully for resistance, since sensitivity can be attenuated in viruses released by human cells after initial cell-cell spread (13).

Our recent interest in the replication of FeLV in human cells was fueled by the desire to use these agents as insertional mutagens and gene discovery tools in human cancer cells in a manner similar to that of murine leukemia viruses (MLVs) in their natural host (14, 15). In support of this goal, the ability of FeLV to integrate preferentially at strong promoters and enhancers is also manifested in human cells infected *in vitro* (16). However, it became apparent from our initial studies that the resistance of human cells to FeLV is a multifaceted phenomenon that is likely to be relevant to cross-species transfer, motivating us to conduct a deeper study.

FeLVs are a family of viruses that frequently occur as phenotypic mixtures in infected cats (17, 18). Three major subgroups, which use different receptors to enter feline host cells, have been described (17). Virtually all natural isolates contain a subgroup A FeLV component (FeLV-A) that enters via the thiamine transporter THTR1 (19). This is the major horizontally transmitted form of FeLV in cats but has been considered unlikely to be zoonotic due to its low affinity for the human receptor homologue, hTHTR1 (19). Many field isolates of FeLV consist of a mixture of FeLV-A and FeLV-B, the latter arising by recombination and acquisition of envelope gene sequences from endogenous FeLV-related sequences (20). FeLV-B isolates can replicate efficiently in at least some cultured human cells with no cytopathic effect (6, 7, 17). Moreover, FeLV-B strains enter via the widely expressed phosphate transporter Pit-1 and/or the related transporter Pit-2 (21). Another human-cell-tropic variant is FeLV-C, which is generated by mutation of the receptor-binding domain of Env (22), facilitating entry through the heme transporter FLVCR1 or FLVCR2 (23, 24). However, FeLV-C isolates are rare in nature (∼1% of isolates) (17) and presumably short-lived, due to their propensity to induce rapidly fatal aplastic anemia (25). A further FeLV envelope variant that has been described is the potently immunosuppressive FeLV-T, but this is also acutely pathogenic and is stringently host-specific; its complex entry requirements include a truncated envelope protein derived from endogenous FeLV-related proviruses that is released by normal feline lymphoid cells (26, 27). Taken together, these biological properties implicate FeLV-B strains as the most likely candidates for zoonotic spread.

While the study of HIV and other primate lentiviruses has identified many factors that control host range by restricting replication (28), the gammaretroviruses have been less well studied in this regard. MLVs have been shown to be highly sensitive to human APOBEC3 cytidine deaminase activity but able to evade murine APOBEC3 (29, 30). The effects of human APOBEC3 activity on FeLV have not been reported, although the remarkable sequence stability of the commonly transmitted form of FeLV in the domestic cat and its low mutation rate in the infection of other felids strongly suggests that it has evolved to evade feline APOBEC activity (31, 32).

This study confirms FeLV-B as the variant most likely to have zoonotic potential, since virtually all human cells are susceptible, with only limited postentry barriers to infection. The resistance of primary blood cells at a postintegration step and potent APOBEC3 induction of mutations in virions released from hematopoietic cells appear to be significant factors in limiting infectivity for human cells. The possibility that FeLV could evolve to evade these barriers cannot be discounted.

## RESULTS

### Human cells display marked differences in susceptibility to FeLV-B infection.

**FIG 1:**
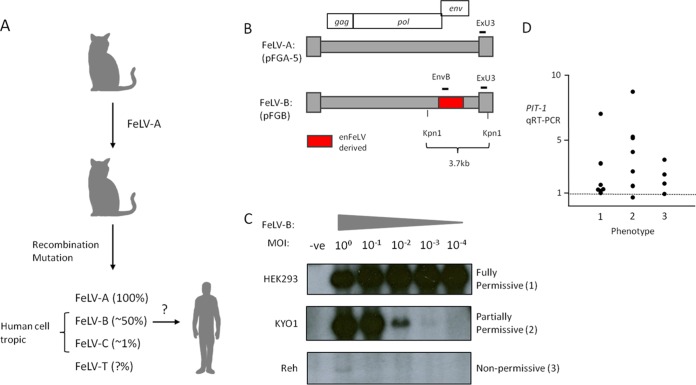
(A) Rationale for focusing on FeLV-B as the most likely zoonotic agent. The percentage of natural isolates containing each FeLV subgroup is given in parentheses. (B) Outline of the molecular structure of FeLV-B proviruses that are derived by recombination between FeLV-A and endogenous (en) FeLV-related proviruses in the feline genome. The hybridization probes (ExU3, EnvB) used to detect FeLV sequences in human cells are indicated. (C) The three basic patterns of susceptibility of cultured human cells to infection with FeLV-B. Cells were exposed to FeLV-B at various multiplicities of infection and were passaged for 14 days before the extraction of high-molecular-weight DNA and analysis of FeLV proviral DNA content by Southern blot hybridization of KpnI-digested DNA with the ExU3 probe. -ve, uninfected control. (D) Relative expression of mRNA encoding the FeLV-B receptor PIT-1 in a panel of cell lines sorted by the pattern of FeLV-B spread (phenotype 1, 2, or 3, as exemplified in panel C).

The rationale for focusing on FeLV-B as the most likely zoonotic variant is presented in Fig. 1A. FeLV-B is the most commonly occurring envelope variant with human cell tropism and is formed by recombination with endogenous FeLV-related sequences (20) (Fig. 1B). To generate FeLV-B virus stocks, the prototypic molecular clone of Gardner-Arnstein FeLV-B was reconstituted by transfection, propagated in HEK293 cells, which release high-titer FeLV-B into cell supernatants (10^5^ to 10^6^ infectious units/ml), and titrated on feline QN10S cells (33). In initial experiments, it was noted that human cells in culture were invariably susceptible to virus infection, but with markedly different proviral copy numbers as assessed by Southern blotting or quantitative PCR. This phenomenon was explored further in a dilution/spreading assay where cells were exposed at decreasing multiplicities of infection (MOI) (from 1 to 10^−4^) and were cultured for 2 weeks to allow virus spread before the harvest of cells and analysis of FeLV proviral DNA by Southern blot hybridization. The detection of an internal 3.7-kb genome fragment provides a quantitative estimate of FeLV DNA content, as shown in Fig. 1C. Three distinct patterns were noted. HEK293 cells are permissive, and FeLV-B spread rapidly to saturation after infection at a low MOI. At the other extreme, the nonpermissive B-cell leukemia cell line Reh showed detectable proviral DNA only at an MOI of 1. The myeloid leukemia cell line KYO-1 showed an intermediate, partially permissive pattern, with the copy number diminishing in proportion to the infectious challenge dose. Quantitative PCR analysis of DNA from cells infected at an MOI of 1 indicated 30-fold-lower levels of FeLV DNA in Reh cells (∼0.3 copy/cell) than in semipermissive KYO-1 cells, which displayed levels similar to those in permissive controls (∼10 copies/cell).

As shown in Table 1, the infection/spread assay revealed full permissiveness (phenotype 1) for the replication of FeLV-B in 7 of 11 human cancer cell lines selected from the NCI-60 cancer cell panel (https://dtp.cancer.gov/discovery_development/nci-60/cell_list.htm). Smaller numbers of cancer cell lines were partially permissive (phenotype 2) or nonpermissive (phenotype 3), with no obvious relationship to the level of expression of receptor PIT-1 mRNA as measured by quantitative reverse transcription-PCR (qRT-PCR), which was in almost all cases higher than that for the permissive HEK293 cell control) (Fig. 1D).

**TABLE 1:** Differential susceptibilities of human cells to infection with FeLV-B

Phenotype^*a*^	Cell line^*b*^	Origin
Fully permissive	HEK293	Adenovirus-transformed immortalized human embryonic kidney cells
	MCF7*	Mammary carcinoma
	MDA-MB-231*	Mammary carcinoma
	HT29*	Colon carcinoma
	SW-620*	Colon carcinoma
	A549*	Lung carcinoma
	DU-145*	Prostate carcinoma
	PC-3*	Prostate carcinoma
	HT-1080	Fibrosarcoma
	TKCC6	Pancreatic carcinoma
	TKCC10	Pancreatic carcinoma
	TKCC27LO	Pancreatic carcinoma
	MiPaCa2S	Pancreatic carcinoma
	Panc10.02	Pancreatic carcinoma
	IMR90	Nonimmortalized lung fibroblasts
	HaCAT	Immortalized skin keratinocytes
Partially permissive	KYO-1	Myeloid leukemia
	CD34^+^	Cord blood hematopoietic precursor
	HeLa*	Cervical carcinoma
	TKCC7	Pancreatic carcinoma
	TKCC9	Pancreatic carcinoma
	TKCC14	Pancreatic carcinoma
	TKCC15LO	Pancreatic carcinoma
	MALME3M*	Melanoma
	Hs68	Primary foreskin fibroblasts
Nonpermissive	Reh	B-cell leukemia
	UACC-62*	Melanoma
	PEO1	Ovarian carcinoma
	OVCAR-4*	Ovarian carcinoma
	RCC4	Renal carcinoma

a Defined according to a dilution/spread assay (Fig. 1C).

b Asterisks indicate NCI-60 panel cell lines.

While spreading phenotype 1 was observed in multiple cancer cell lines, we noted that Hs68 primary foreskin fibroblasts were more restrictive. To explore the possibility that phenotype 1 is a feature of long-established cancer cell lines, we extended our study to a panel of recently established pancreatic cancer cell lines (34) as well as other nontransformed cells, including nonimmortalized CD34^+^ cord blood cells, lung fibroblasts (IMR90), and immortalized but nontransformed skin keratinocytes (HaCAT). Where cell numbers were limiting (e.g., for CD34^+^ cells), FeLV DNA content was analyzed by quantitative PCR. Pancreatic cancer cell lines displayed either the fully or the partially permissive phenotype, while the partially permissive phenotype was also observed in CD34^+^ cord blood cells. The fully permissive phenotype was observed in HaCAT and IMR90 cells, indicating that permissiveness is not simply a consequence of oncogenic transformation. It is also noteworthy that we observed no evidence of cytopathic effect or significant growth alteration in any of the FeLV-B-infected cell cultures, indicating that restricted viral spread was not due to loss of cell viability or interference with cell division. In contrast, FeLV-C variants have marked cytopathic effects in some cells (35).

### Human hematopoietic cells display a partially permissive or nonpermissive phenotype.

**FIG 2:**
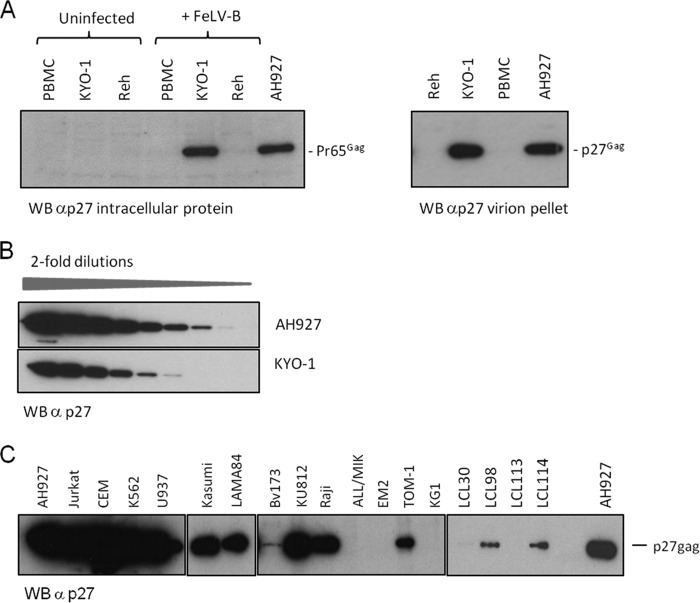
Human hematopoietic cells display two patterns of susceptibility to FeLV-B replication. (A) (Left) Western blot (WB) analysis of the intracellular Gag precursor protein (Pr65^Gag^) in partially permissive (phenotype 2) FeLV-B-infected KYO-1 cells shows expression levels similar to those in fully permissive feline fibroblasts (AH927). In contrast, expression of Pr65^Gag^ is barely detectable in nonpermissive Reh cells and human PBMCs. (Right) This pattern is reflected in the levels of virus particle release, which are similar in partially permissive KYO-1 cells and permissive AH927 cells, as illustrated by Western blot analysis of pelleted virion preparations from equal volumes of culture medium. (B) Results of a separate experiment where Western blot analysis of serial 2-fold dilutions of virions released from AH927 and KYO-1 cells again showed levels of particle release from KYO-1 cells within a range similar to that for AH927 cells. (C) Western blot analysis of pelleted virions harvested from cells 14 days after infection with FeLV-B. Equal volumes of cell supernatant (2 ml) were sampled 24 h after medium change and were processed as described in Materials and Methods.

The permissive spreading phenotype in multiple cancer cell lines is associated with the release of high-titer FeLV at levels comparable to those in feline fibroblast controls. To explore the basis of the restricted replication phenotypes in greater depth, we examined the expression of intracellular viral antigen and virion production in representative cell lines (KYO-1 and Reh) that were exposed to FeLV-B at MOI of >1 and cultured for 2 weeks to allow viral spread. To explore the hypothesis that hematopoietic cells are more widely resistant than other cell types, we extended this analysis to a wider panel of hematopoietic cell lines as well as primary human peripheral blood mononuclear cells (PBMCs). As shown in Fig. 2A, infected KYO-1 cells express the FeLV precursor polyprotein Pr65^Gag^ at levels similar to those in permissive feline fibroblast cells (AH927), while Reh cells and PBMCs produce low or undetectable levels. A similar divergence was noted when virion production was analyzed. At 14 days postinfection, partially permissive KYO-1 cells showed sustained release of virions at levels similar to those in permissive feline cells, while virion release by Reh cells and PBMCs was low to undetectable (Fig. 2A). In a separate experiment, the virtual equivalence of virion protein release was illustrated by serial dilution of pelleted virion preparations prior to Western blot analysis (Fig. 2B). As shown in Fig. 2C and Table 2, extending this analysis to a wider panel of hematopoietic cell lines showed that 9 of 13 leukemia cell lines displayed a KYO-1-like phenotype with sustained high-level release of virions, while the other 4 resembled Reh cells, with very low to undetectable levels of virus release. The level of permissiveness showed no clear pattern with regard to the type of leukemia (B cell, T cell, myeloid, erythroleukemia) or the cytogenetic features of the leukemia cell lines. PBMCs from four separate donors and a series of four Epstein-Barr virus (EBV)-immortalized lymphoblastoid cell lines (LCLs) showed a low to nonpermissive phenotype in this assay. An assay for infectious FeLV released by these cells revealed a similar pattern. Not surprisingly, cells with low virion release did not release significant levels of infectious FeLV. However, virus released at high levels from partially permissive lines showed very marked differences in specific infectivity, which was 4- to 10^4^-fold lower than that of virus released from permissive feline cells. The CEM cell line and its derivative CEMSS (36) were the most permissive lines tested, while Raji and LAMA84 cells released the least infectious virions (Table 2).

**TABLE 2:** Human hematopoietic cell lines release FeLV-B virions with markedly different specific infectivities

Cell line	Origin^*a*^	Level of virion release^*b*^	FeLV titer (FFU/ml)^*c*^
HEK293	Human embryonic kidney cells	High	10^5^–10^6^
AH927	Immortalized feline fibroblasts	High	4 × 10^5^
HT1080	Fibrosarcoma	High	4 × 10^5^
KYO-1	CML (blast crisis), t(9;22)	High	3 × 10^3^
CEM	T-cell acute lymphoblastic leukemia	High	4 × 10^4^
CEMSS	T-cell acute lymphoblastic leukemia	High	6 × 10^4^
Jurkat	T-cell acute lymphoblastic leukemia	High	4 × 10^2^
K562	Erythroleukemia (CML blast crisis), t(9;22)	High	10^3^
Kasumi	Acute myeloblastic leukemia	High	3.5 × 10^2^
LAMA84	Basophilic (CML blast crisis), t(9;22)	High	1.3 × 10^1^
Raji	B-cell (Burkitt lymphoma), t(8;14)	High	3 × 10^1^
TOM-1	B-cell acute lymphocytic leukemia, t(9;22)	High	1.6 ×10^1^
KU812	Basophilic/erythroleukemia (CML blast crisis), t(9;22)	High	2.3 × 10^3^
Reh	Pre-B-cell acute lymphocytic leukemia, t(12;21)	Low	2 × 10^0^
bv173	Undifferentiated blast, t(9;22)	Low	2
ALL/MIK	Pre-B-cell acute leukemia, t(9;22)	Low/negative	5 × 10^1^
EM2	Myeloblast (CML), t(9;22)	Low/negative	2
KG-1	Acute myelogenous leukemia (M1)	Low/negative	0
PBMC (*n* = 4)	Normal peripheral blood monocytic cells	Negative	0
LCL (*n* = 4)	EBV-immortalized B-lymphoblastoid cell lines	Low	0

a CML, chronic myeloid leukemia.

b Measured at 14 days after infection with FeLV-B at an MOI of 1 (Fig. 2C).

c Infectious FeLV titer measured on QN10 (S^+^ L^−^) cells. FFU, focus-forming units.

### The block to FeLV-B replication in nonpermissive cells occurs after reverse transcription.

**FIG 3:**
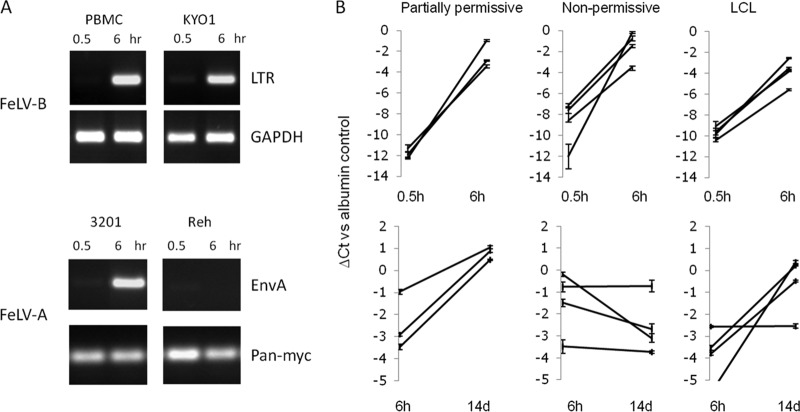
Permissive and nonpermissive human cells support FeLV-B entry and early proviral DNA synthesis at similar levels. (A) Initial studies using standard PCR with primers based on FeLV LTR or envelope sequences showed marked increases in proviral DNA levels between 0.5 and 6 h after the exposure of cells to FeLV-B at an MOI of 1. Because no PIT-1-negative cell lines were identified, a receptor-negative control was generated for this assay by comparing the FeLV-A infection of permissive feline cells (3201 cells) and of Reh cells, which lack detectable expression of mRNA for the human homologue of FeLV-A receptor THTR1. Loading controls used human glyceraldehyde-3-phosphate dehydrogenase (GAPDH) or pan-Myc primers, which efficiently detect human and feline DNA. (B) A wider analysis of FeLV-B infection of human hematopoietic cell lines. Proviral DNA levels were analyzed by quantitative PCR. The line graphs show changes in FeLV DNA concentrations (detected by EnvB primers) relative to those of a housekeeping gene control (β2-microglobulin) between 0.5 and 6 h (top) or between 6 h and 14 days (bottom) after infection. The *y* axis shows the threshold cycle (*C_T_*) value relative to that of the β2-microglobulin control (taken as zero). Partially permissive cells were CEM, Jurkat, and Raji cells. Nonpermissive cells were PBMCs (*n* = 2), Reh cells, and ALL/MIK cells. Lymphoblastoid cell lines were LCL30, LCL98, LCL113, and LCL114.

To explore the basis of the nonpermissive phenotype in greater detail, we examined the accumulation of proviral DNA after the exposure of cells to FeLV-B at an MOI of 1. Notably, nonpermissive PBMCs accumulated levels of FeLV DNA similar to those observed in partially permissive, high-virion-release KYO-1 cells (Fig. 3A). This was not due to detection of preexisting DNA in virions, since only very low levels were detected after the 30-min adsorption period, with significant increases by 6 h. To confirm that this accumulation is a receptor-dependent process, we compared FeLV-A infection of permissive feline lymphoma cells (3201 cells) to that of Reh cells, which do not express detectable mRNA for the human receptor homologue THTR1. This control was chosen because no PIT-1-negative cell lines were identified. The lack of accumulation of proviral DNA in the receptor-negative cells supports the hypothesis that this process is the result of receptor-mediated entry. The efficient detection of newly synthesized DNA with either long terminal repeat (LTR) or envelope gene primers suggests that complete proviral sequences are present, with no bias toward “strong-stop” products (37).

More-extensive analysis of postadsorption amplification of proviral DNA by quantitative PCR showed that all cells tested were initially permissive regardless of the later outcome of infection (Fig. 3B). However, comparison of the quantitative change in DNA from the 6-h time point to late infection (14 days) revealed a stark contrast between the semipermissive, high-virion-release cells (e.g., CEM, Raji, and Jurkat cells), where copy numbers increased over time, and the low-virion-output, nonpermissive cells (e.g., PBMCs and Reh cells), which showed unchanged or decreasing levels of proviral DNA. Intriguingly, EBV-immortalized cell lines mainly fell into the partially permissive category in this assay, suggesting that significant viral spread had occurred in these cells, while day-14 analysis revealed only low levels of virion release with no detectable infectivity. Again, we noted no cytopathic effect or changes in cell growth in these experiments.

### Integrated FeLV-B DNA persists in nonpermissive human PBMCs.

**FIG 4:**
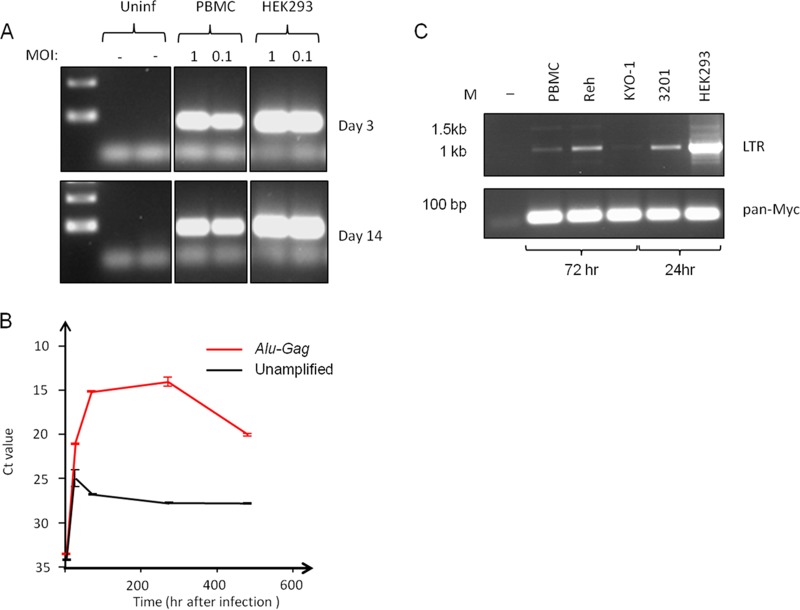
Persistence of integrated FeLV-B DNA in human PBMCs. (A) Semiquantitative PCR reveals dose-dependent persistence of FeLV DNA in human PBMCs at 14 days postinfection. Fully permissive HEK293 control cells show stable levels by 3 days postinfection. (B) Results of a quantitative PCR time course analysis for FeLV sequences in PBMC DNA, with and without a preamplification step using primers to FeLV Gag and consensus human Alu sequences. The threshold cycle (*C_T_*) value is inversely proportional to the DNA content. (C) Results of semiquantitative PCR for LTR circles in FeLV-B-infected cells 3 days (PBMC, Reh, and KYO-1 cells) or 24 h (3201 and HEK293 cells) after infection. While single and double LTR circle forms are readily detected in productively infected HEK293 and 3201 cells, they are barely detectable in Reh cells and PBMCs relative to levels in permissive HEK293 control cells. The loading control was provided by PCR amplification with conserved Myc primers (pan-Myc).

Despite the lack of proviral DNA amplification, we noted that FeLV-B DNA was readily detectable in PBMCs at similar levels between 3 and 14 days after initial infection (Fig. 4A). This stable association suggested that FeLV-B DNA may be integrated into PBMCs, and this hypothesis was tested further by quantitative PCR after preamplification with a FeLV Gag primer and a consensus human Alu repeat sequence (Fig. 4B). The greatly enhanced detection of FeLV DNA after Alu-Gag preamplification indicates that much of the DNA is in an integrated form. The possibility that FeLV DNA persists in an unintegrated form was also tested by PCR for circular forms generated by ligation in the nucleus. LTR circles were barely detectable in PBMCs and Reh cells by 3 days postinfection. Positive controls for this assay were early (24-h) samples from productively infected 3201 and HEK293 cells, where single and double LTR circles were detected (Fig. 4C). Cloning and sequencing confirmed the identity of the single-LTR form (not shown).

### Partially permissive cells display G-to-A hypermutation that correlates with A3G mRNA expression.

**FIG 5:**
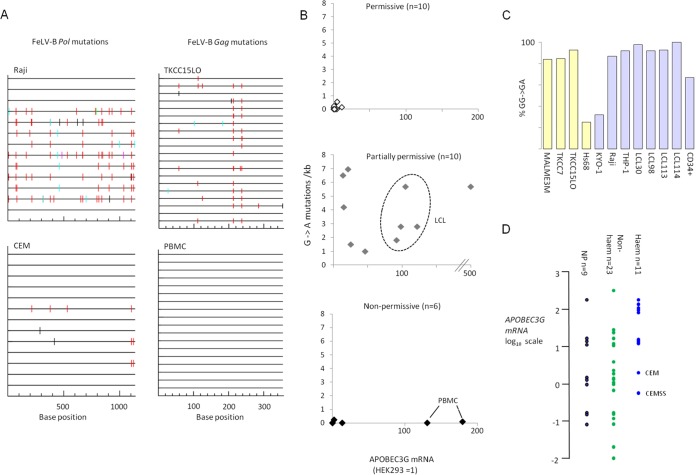
Hypermutation of the FeLV-B genome and APOBEC3G mRNA expression in human cells. FeLV genomic sequences (segments of Gag and Pol) were cloned by PCR from infected human cells, and individual templates were sequenced and compared to the reference input virus (pFGB clone). APOBEC3G mRNA expression was determined in the same cells (prior to infection) by quantitative real-time PCR (with SYBR green). (A) Representative plots of hypermutation visualized by the online HYPERMUT program (www.hiv.lanl.gov/content/sequence/HYPERMUT/hypermut.html), where sequence changes relative to the reference FeLV-B genome are color coded (red, GG→AG; cyan, GA→AA; green, GC→AC; magenta, GT→AT; black, non-G→A). (B) X/Y plots of G→A mutation (per kilobase) against APOBEC3G mRNA levels (where the level in HEK293 cells is taken as 1) with cell lines sorted according to FeLV restriction phenotype. Results for LCLs, which are discordant by virtue of their low levels of infectious virion release (Table 2) but postinfection accumulation of proviral DNA (Fig. 3), are enclosed by a dashed oval. (C) Percentage of G→A mutations that conform to the A3G signature (42) for all cell lines in which significant levels of mutations were detected. Blue-gray bars, hematopoietic cells; yellow bars, nonhematopoietic cells. (D) Relative levels of APOBEC3G mRNA (on a log_10_ scale, with the level in HEK293 cells taken as 1) for all the cell lines tested, sorted into hematopoietic (blue circles) and nonhematopoietic (green circles) cell lines. Nonpermissive cells (nonspreading, with low virion release) are represented by black circles.

To explore the hypothesis that APOBEC cytidine deaminase activity is involved in FeLV growth restriction, we analyzed proviral DNA after PCR amplification of genome fragments from infected cells. These sequences were compared to the index FeLV-B (pFGB) sequence for evidence of characteristic G→A mutations that are induced during reverse transcription (38). We found evidence of extensive hypermutation of FeLV replicating in human cells at rates as high as 7 mutations/kb. This mutation rate is much higher than the G→A mutation rate reported for FeLV replicating in cats *in vivo* (0.1/kb) (39), indicating that FeLV, like MLV (30), is able to evade this restriction factor only in its natural host. FeLV strains typically encode a glycosylated Gag precursor open reading frame (40), and this is intact in the Gardner-Arnstein FeLV-B molecular clone. However, FeLV, like MLV, is sensitive to heterologous human APOBEC3 activity (41). As shown in Fig. 5 with the examples of the Burkitt lymphoma cell line Raji and the pancreatic cancer cell line TKCC15LO, mutational footprints indicated that the predominant signature was GG→GA, suggesting that APOBEC3G (A3G) is responsible (42). Moreover, expression of A3G mRNA showed a positive correlation with mutational activity when all cell lines were compared (*R* = 0.22; *P* = 0.01). The results are collated in Fig. 5B and are broken down according to the permissive (phenotype 1), partially permissive (phenotype 2) (high virion release), or nonpermissive (phenotype 3) (low virion release) phenotype. Not surprisingly, permissive cells showed low A3G expression and low mutational activity, while partially permissive, high-virion-release cells showed high A3G expression and mutational activity. In contrast, nonpermissive cells showed no evidence of hypermutation, indicating that a nonmutational mechanism of resistance is operative in these cells. Moreover, a lack of mutation in PBMCs was observed despite very high levels of A3G, suggesting that the proviral DNA detected was the result of initial reverse transcription rather than secondary spread. The four LCLs showed significant levels of hypermutation (Fig. 5B, center, dashed oval), supporting their grouping with the partially permissive cells on the basis of proviral DNA accumulation (Fig. 3).

Analysis of all G→A mutations in the cell lines with significant levels of hypermutation (Fig. 5C) indicated that A3G is the major, though not the sole, effector of G→A mutations in the FeLV genome during replication in human cells. Notably, the two cell lines with divergent signatures (Hs68 and KYO-1) displayed high levels of APOBEC3F mRNA (not shown). The expression levels of A3G mRNA detected ranged over several log units but were notably higher in cells of hematopoietic origin (Fig. 5D). The CEM cell line and it derivative CEMSS showed the lowest levels among hematopoietic cell lines, in agreement with their low mutation rates and high levels of release of relatively noninfectious FeLV virions (Table 2). High levels of A3G mRNA relative to that in the HEK293 control were also associated with restricted FeLV spread in nonhematopoietic cells, but the majority of these lines showed low expression and a fully permissive phenotype. However, as indicated in Fig. 5D, the nonpermissive phenotype (nonspreading phenotype 3 [Table 1] and low proviral DNA accumulation [Fig. 3]) shows no relationship with A3G expression or the mutation rate. This finding, taken together with evidence that this phenotype arises from a postintegration block to incoming virus, suggests that this is an A3G-independent barrier.

## DISCUSSION

FeLV-B strains are human-cell-tropic variants that are generated frequently during FeLV infection *in vivo* (20, 43, 44) and are therefore of prime interest with respect to the zoonotic potential of FeLV. This study demonstrated fully permissive or partially permissive infection in a broad range of cancer-derived cell lines and in nontransformed keratinocytes and lung fibroblasts. Cell lines of hematopoietic origin were generally less permissive and were assigned to two discrete groups based on high or low FeLV protein expression and virion release. Primary PBMCs and some EBV-transformed LCLs were nonpermissive with respect to these measures (Fig. 1C and 2; Table 2). While a spectrum of phenotypes was observed, this study revealed apparently universal susceptibility of human cells to initial infection with FeLV-B, with no evidence of a significant preintegration block to infection (Fig. 4); indeed, the early steps of reverse transcription and integration appear to be unimpaired in nonpermissive cells (Fig. 3 and 5A to C). However, at least two postintegration blocks to FeLV-B replication appear to be operative in human cells. One block is clearly mediated by the APOBEC system, as indicated by the high rates of G→A mutations in FeLV in leukemia cell lines that release abundant viral particles with low infectivity and in nonhematopoietic cancer cell lines that display a partially permissive phenotype with respect to viral spread (e.g., Raji and TKCC15LO cells, respectively) (Table 2; Fig. 3B and 5). The predominant mutational signature was indicative of A3G in most cases, and we noted a weak correlation between A3G mRNA expression and mutational activity across a panel of cell lines (Fig. 5). This observation does not exclude the possibility that A3G also inhibits by nonmutational mechanisms, such as inhibition of reverse transcription (45).

The profound block to FeLV mRNA and protein expression in primary PBMCs (Fig. 2) and to virus release from these cells occurred without detectable mutation, despite the expression of high levels of A3G mRNA (Fig. 5), indicating a restriction that is A3G independent. APOBEC3 proteins have also been reported to block incoming murine retroviruses, and it appears that ectopic expression of A3A in mice can do so by a nonmutational mechanism (41). However, as far as we are aware, there is no evidence that APOBEC restriction can operate on incoming viruses after integration. Potential mediators of postintegration suppression of incoming viruses include epigenetic regulators, such as those that are abundantly expressed in embryonal stem cells, and restrict the expression of exogenous and endogenous retroelements (46). The possibility that the resistance of PBMCs to FeLV reflects such a general resistance mechanism against gammaretrovirus zoonosis merits further study. In this regard, we note reports of profound restriction of xenotropic murine leukemia virus-related virus (XMRV) growth in human PBMCs, which showed evidence of replication with G→A mutations only at extremely high levels of viral input (47).

With regard to relevance to zoonotic resistance, a key question is whether the widespread susceptibility to FeLV replication observed in human cells reflects the phenotype of the tissue of origin or a loss-of-function phenomenon due to oncogenic transformation. The strongly restrictive phenotype of PBMC cultures, which contain a rich variety of primary cell types, suggests that restriction is likely to represent an important *in vivo* barrier. While the relative susceptibility of leukemia cell lines may be due to loss of function, the similar phenotype observed in primary CD34^+^ cells (Table 1) suggests that this may instead reflect an immature progenitor phenotype. Moreover, many nonhematopoietic cell lines proved to be highly permissive for transcription, virion assembly, and release of FeLV-B particles, with no evidence of APOBEC-mediated mutations. Observation of this phenotype in IMR90 nontransformed lung fibroblasts, for example, which retain the capacity for replicative senescence (48), as well as in HaCAT skin keratinocytes, shows that susceptibility to infection can occur in the absence of oncogenic transformation (Table 1). Moreover, the earliest description of productive FeLV replication in human cells was based on infection of HEL2000 primary lung fibroblasts (6). In the case of transformed cells, it is difficult to discern whether the relative susceptibility to FeLV represents a normal cellular state relevant to potential zoonosis. While very-high-level A3G expression and mutational activity were observed in the TKCC15LO pancreatic cancer cell line (Fig. 5), this may not reflect the normal phenotype of pancreatic tissue. Upregulated A3G has been postulated to play a direct oncogenic role in some pancreatic cancers (49).

The susceptibility of human cancer cells to spreading FeLV-B replication provides a potentially instructive parallel with the mobilization and frequent reinsertion of L1 retrotransposons in >50% of human cancers (50); high levels were noted in lung, colon, prostate, and breast cancers, while the data sets for other tumor types showed few if any new insertions in renal carcinomas and melanomas. These findings parallel the present observations in that colon, lung, and breast cancer cell lines were fully permissive to FeLV infection, but renal cancer cells and one melanoma line displayed a strong early block to FeLV replication, with no evidence of APOBEC-mediated mutations (Table 1; Fig. 5B). A further intriguing parallel is provided by a recent study of XMRV pathogenesis in macaques, where intravenous inoculation of high-titer virus led to the dissemination and persistence of viral protein expression, with the highest levels in lung, colon, and prostate tissues (51). Taken together, these observations suggest that susceptibility to infection may be the default state in some primary tissues. Moreover, susceptibility to FeLV may provide a useful marker to aid in the identification of further factors that confer tissue-specific susceptibility to genome instability in cancer as well as resistance to incoming retroviruses.

Can the risk of zoonotic infection with FeLV be discounted? Despite control measures, including vaccination, that have reduced the prevalence of FeLV in some parts of the world, this virus family remains one of the most important pathogens of cat species worldwide (52). Moreover, the limited barriers protecting human cells against FeLV infection contrast with the plethora of restriction factors that protect against the cross-species transmission of primate lentiviruses, where recent jumps have occurred (53). The successful transfer of other gammaretroviruses that use PIT-1 as an entry receptor from rodents to primates (5) argues that future adaptation of FeLV for zoonotic spread is not beyond the repertoire of this virus family. The possibility of human exposure to high doses of FeLV analogous to those employed in recent primate challenge experiments with XMRV (51, 54) is extremely remote; however, it may be interesting to revisit the apparent lack of adaptive immune responses in exposed individuals, e.g., veterinary workers (10), using more-sensitive techniques.

## MATERIALS AND METHODS

### Virus stocks.

FeLV-B stocks were generated by transfection of an infectious molecular clone (pFGB) (55) and propagation in the feline fibroblast cell line AH927 or HEK human embryonic kidney cells as described previously (56). For FeLV-A stocks, HEK293 cells were transfected with pFGA-5 (20) to avoid possible recombination and generation of FeLV-B (57). FeLV infectivity was determined by titration on S^+^ L^−^ QN10 cells (33).

### Cells.

Cell lines were derived from curated frozen stocks maintained in our laboratory (Jurkat, K562, CEM, Reh, Kasumi, U937, MCF-7) or by colleagues in the Glasgow Cancer Research UK Centre, including A. Biankin (pancreatic cancer TKCC series) and T. Holyoake (chronic myeloid leukemia lines KYO-1, BV173, KU812, LAMA84, and EM-2; B-cell acute lymphoblastic leukemia lines ALL/MIK and TOM-1). IMR90 fibroblasts were provided by P. Adams (48), while EBV-transformed lymphoblastoid cell lines (LCLs) were obtained from R. Jarrett, and HaCAT keratinocytes were provided by S. Graham. CEMSS cells were kindly provided by M. Malim (King's College, London, United Kingdom). CD34^+^ cells were purified by positive selection using anti-CD34-conjugated magnetic microbeads (catalog no. 130-046-703; Miltenyi Biotec Inc.) from cord blood samples generously provided by the Children's Cancer and Leukemia Group (CCLG). Primary blood mononuclear cells were obtained from Cambridge Bioscience (1 sample) and the Scottish National Blood Transfusion Service (3 samples).

### Dilution/spreading assay and Southern blot analysis.

A total of 4 × 10^5^ KYO-1 cells or Reh cells or 5 × 10^4^ HEK293 cells were plated per well in 6 wells of a 12-well plate the day before infection. FeLV-B was harvested from subconfluent virus-producing HEK293 cells, filtered through 0.45-μm filters, and serially diluted 1:10 in RPMI 1640 medium with 10% fetal calf serum (FCS). A 0.5-ml volume of filtered diluted virus was added per well, or medium alone was added to the control well, for 2 h. Cells were then fed with fresh medium and were expanded as normal for 14 days. Genomic DNA was extracted from phosphate-buffered saline (PBS)-washed, pelleted cells using a Qiagen DNeasy blood and tissue kit. Aliquots (15 μg) of genomic DNA were digested overnight with KpnI, separated on a 0.8% agarose Tris-acetate-EDTA (TAE) gel, transferred to a Hybond N membrane (Amersham) in 20 × SSC (1× SSC is 0.15 M NaCl plus 0.015 M sodium citrate), and hybridized to FeLV ExU3 as described previously (21).

### Quantitative real-time RT-PCR.

RNA was extracted from cultured cells using a Qiagen RNeasy minikit, and cDNA was synthesized from 1 μg RNA using a Qiagen QuantiTect reverse transcription kit. Aliquots (12.5 ng) of cDNA were amplified in triplicate using Power SYBR green PCR master mix (Thermo Fisher Scientific) and Qiagen QuantiTect primer assays for human hypoxanthine phosphoribosyltransferase (HPRT) (QT00059066), APOBEC3G (QT00070770), SLC20A1 (PIT-1) (QT00028763), or SLC19A2 (THTR1) (QT00007847) on an ABI 7500 real-time PCR system. Quantification was carried out relative to HEK293 cells, and values were normalized to HPRT expression.

### Semiquantitative DNA PCR.

One hundred-nanogram aliquots of genomic DNA were amplified in Reddy Mix (Thermo Fisher Scientific) using primers FeLV envA F (5′-TAAAACACGGGGCACGTTAC-3′) and FeLV envA R (5′-GGAGGTGGGCTTCCACCAAG-3′), FeLV envB F (5′-ATGTGATCAGCCTATGAGGA-3′) and FeLV envB R (5′-CACTAGCTCCCGTTGTCGAG-3′), FeLV LTR F (5′-TAGCTGAAA CAGCAGAAGTTTCAAG-3′) and FeLV LTR F R (5′-GGAAGGTCGAACTCTGGTCAAC-3′), and Myc F (5′-CCAACAGGAACTATGACCTCG-3′) and Myc R (5′-GTAGAAGTTCTCCTCCTCGTC-3′). Samples were denatured at 94°C for 3 min and were then subjected to 35 cycles of 94°C for 30 s, 60°C for 30 s, and 72°C for 30 s. Amplification products were separated by electrophoresis on 2% agarose-TAE gels and were visualized by ethidium bromide staining.

### DNA quantitative PCR.

Genomic DNA was extracted by a Qiagen DNeasy minikit, and 20-ng aliquots were amplified using primers for human β2 microglobulin (F, 5′-GGAATTGATTTGGGAGAGCAT-3′; R, 5′-CAGGTCCTGGCTCTACAATTTACTAA-3′), primers FeLV U3-LTR F (5′-TAGCTGAAA CAGCAGAAGTTTCAAG-3′) and FeLV U3-LTR R (5′-GGAAGGTCGAACTCTGGTCAAC-3′), and primers envB F (5′CGGTATCCCGGCAAGTAATG-3′) and envB R (5′-GGTTTTTGATCAGGCAGGACTAGA-3′) in Power SYBR green PCR master mix. Amplification products were detected on an ABI 7500 real-time PCR system. Samples were normalized to β2 microglobulin.

### Western blotting.

PBS-washed, pelleted cells were lysed in whole-cell lysis buffer and protein concentrations measured by a Bio-Rad protein assay. Virion lysates were prepared from 2 ml precleared virion supernatant by centrifugation in a Beckman TL100 benchtop ultracentrifuge for 2 h at 37,000 rpm and 4°C. Virion pellets were resuspended in 75 μl lysis buffer, and an equal volume (13 μl) of virion protein or 10-μg aliquots of cellular protein were separated on 4-to-12% NuPAGE Novex Bis-Tris gels in NuPAGE morpholinepropanesulfonic acid (MOPS) SDS buffer and were transferred to Hybond ECL membranes (Amersham) in NuPAGE transfer buffer. Following overnight blocking in 5% milk in Tris-buffered saline–Tween 20 (TBST), the filter was exposed to a 1:500 dilution of an anti-p27^Gag^ monoclonal antibody (VPG19.1; a gift of Brian Willett), and detection was carried out with the ECL detection reagent (GE Healthcare).

### Hypermutation analysis.

FeLV *gag* or *pol* fragments were generated by amplification of 100-ng aliquots of infected-cell genomic DNA in Reddy Mix PCR master mix (Thermo Fisher) using primers FeLV p27gag F (5′-CCCAGTGGCCCTAACTAACC-3′) and R (5′-GCTGGCGTTTCCTCTTTCC-3′) or FeLV Pol F (5′-GCAACCGGTAAGGTGACTC-3′) and Pol R (5′-TTCAAAGGGTTTGGTGATATCTG-3′). DNA was denatured at 94°C for 3 min and was then put through 35 cycles of 94°C for 30 s, 60°C for 30 s, and 72°C for 30 s. Products were purified through Qiagen QIAquick PCR purification columns and were then cloned into pCR2.1-TOPO (Invitrogen), transformed into TOP10 competent cells, and plated onto Amp X-Gal (5-bromo-4-chloro-3-indolyl-β-d-galactopyranoside) agar plates. White colonies were cultured and plasmid DNA extracted using Qiaprep spin preps, and DNA was sequenced from M13 F and M13 R primers by Source BioScience and was examined for mutations relative to the pFGB molecular clone using Hypermut, version 2.0.

### Alu-Gag PCR.

Primary PCR on 50 ng genomic DNA was performed in Platinum *Taq* DNA polymerase (Invitrogen) using 10 μM primers AluF (5′-GCCTCCCAAAGTGCTGGGATTACAG-3′) and FeLV gag R (5′-ATAGGGAGGTGGCTCTTCTG-3′) at 95°C for 2 min, followed by 40 cycles of 95°C for 15 s, 50°C for 15 s, and 72°C for 3 min 30 s. Nested PCR was carried out on 4 μl primary PCR product or 4 ng unamplified DNA using primers FeLV RU5 F (5′-TCTTTGCTGAGACTTGACCG-3′) and FeLV RU5 R (5′-ACTAGGTCTTCCTCGGCGAT-3′) and probe FeLV RU5 (5′-FAM-TGCATCTGACTCGTGGTCTC-TAMRA-3′) using Takyon LowRox Probe Mastermix dTTP blue (UF-LPMT-BO101) (Eurogentec) and settings of 50°C for 2 min and 95°C for 3 min, followed by 40 cycles of 95°C for 10 s and 60°C for 60 s on an ABI 7500 real-time PCR system.

### LTR circle detection.

Aliquots (100 ng) of genomic DNA from 3-day (PBMCs, Reh and KYO-1 cells) or 24-h (3201 and HEK293 cells) FeLV-infected cells were amplified in Reddy Mix PCR master mix with primers Circles env F (5′ TAAAACAGCGGCAACAACTG-3′) and Circles gag R (5′-GTCTCCGATCAACACCCTGT-3′) or the positive-control primers pan-Myc 23F (5′-CCAACAGGAACTATGACCTCG-3′) and pan-Myc 96R (5′-GTAGAAGTTCTCCTCCTCGTC-3′). Following an initial denaturation step at 94°C for 3 min, 35 cycles of 94°C for 30 s, 60°C for 30 s, and 72°C for 1 min 30 s were performed. Portions (15 μl) of each 25-μl reaction mixture were separated on 1.4% agarose-TAE gels and were visualized by staining with ethidium bromide.

## References

[1] Lander ES, Linton LM, Birren B, Nusbaum C, Zody MC, Baldwin J, Devon K, Dewar K, Doyle M, FitzHugh W, Funke R, Gage D, Harris K, Heaford A, Howland J, Kann L, Lehoczky J, LeVine R, McEwan P, McKernan K, Meldrim J, Mesirov JP, Miranda C, Morris W, Naylor J, Raymond C, Rosetti M, Santos R, Sheridan A, Sougnez C, Stange-Thomann Y, Stojanovic N, Subramanian A, Wyman D, Rogers J, Sulston J, Ainscough R, Beck S, Bentley D, Burton J, Clee C, Carter N, Coulson A, Deadman R, Deloukas P, Dunham A, Dunham I, Durbin R, French L, ; International Human Genome Sequencing Consortium. 2001 Initial sequencing and analysis of the human genome. Nature 409:860–921. doi:10.1038/35057062. 11237011

[2] Gifford R, Kabat P, Martin J, Lynch C, Tristem M 2005 Evolution and distribution of class II-related endogenous retroviruses. J Virol 79:6478–6486. doi:10.1128/JVI.79.10.6478-6486.2005. 15858031 PMC1091674

[3] Olah Z, Lehel C, Anderson WB, Eiden MV, Wilson CA 1994 The cellular receptor for gibbon ape leukemia virus is a novel high affinity sodium-dependent phosphate transporter. J Biol Chem 269:25426–25431. 7929240

[4] Xu W, Stadler CK, Gorman K, Jensen N, Kim D, Zheng H, Tang S, Switzer WM, Pye GW, Eiden MV 2013 An exogenous retrovirus isolated from koalas with malignant neoplasias in a US zoo. Proc Natl Acad Sci U S A 110:11547–11552. doi:10.1073/pnas.1304704110. 23798387 PMC3710800

[5] Alfano N, Kolokotronis SO, Tsangaras K, Roca AL, Xu W, Eiden MV, Greenwood AD 2016 Episodic diversifying selection shaped the genomes of gibbon ape leukemia virus and related gammaretroviruses. J Virol 90:1757–1772. doi:10.1128/JVI.02745-15. PMC4734002 26637454

[6] Jarrett O, Laird HM, Hay D 1969 Growth of feline leukaemia virus in human cells. Nature 224:1208–1209. doi:10.1038/2241208a0. 5358345

[7] Sarma PS, Huebner RJ, Basker JF, Vernon L, Gilden RV 1970 Feline leukemia and sarcoma viruses: susceptibility of human cells to infection. Science 168:1098–1100. doi:10.1126/science.168.3935.1098. 5441683

[8] Krengel A, Cattori V, Meli ML, Wachter B, Boni J, Bisset LR, Thalwitzer S, Melzheimer J, Jago M, Hofmann-Lehmann R, Hofer H, Lutz H 2015 Gammaretrovirus-specific antibodies in free-ranging and captive Namibian cheetahs. Clin Vaccine Immunol 22:611–617. doi:10.1128/CVI.00705-14. 25809630 PMC4446404

[9] Meli ML, Cattori V, Martinez F, Lopez G, Vargas A, Simon MA, Zorrilla I, Munoz A, Palomares F, Lopez-Bao JV, Pastor J, Tandon R, Willi B, Hofmann-Lehmann R, Lutz H 2009 Feline leukemia virus and other pathogens as important threats to the survival of the critically endangered Iberian lynx (Lynx pardinus). PLoS One 4:e4744. doi:10.1371/journal.pone.0004744. 19270739 PMC2649436

[10] Butera ST, Brown J, Callahan ME, Owen SM, Matthews AL, Weigner DD, Chapman LE, Sandstrom PA 2000 Survey of veterinary conference attendees for evidence of zoonotic infection by feline retroviruses. J Am Vet Med Assoc 217:1475–1479. doi:10.2460/javma.2000.217.1475. 11128537

[11] Rother RP, Fodor WL, Springhorn JP, Birks CW, Setter E, Sandrin MS, Squinto SP, Rollins SA 1995 A novel mechanism of retrovirus inactivation in human serum mediated by anti-alpha-galactosyl natural antibody. J Exp Med 182:1345–1355. doi:10.1084/jem.182.5.1345. 7595205 PMC2192220

[12] Sherwin SA, Benveniste RE, Todaro GJ 1978 Complement-mediated lysis of type-C virus: effect of primate and human sera on various retroviruses. Int J Cancer 21:6–11. doi:10.1002/ijc.2910210103. 75191

[13] Takeuchi Y, Cosset FL, Lachmann PJ, Okada H, Weiss RA, Collins MK 1994 Type C retrovirus inactivation by human complement is determined by both the viral genome and the producer cell. J Virol 68:8001–8007. 7966590 10.1128/jvi.68.12.8001-8007.1994 PMC237263

[14] Huser CA, Gilroy KL, de Ridder J, Kilbey A, Borland G, Mackay N, Jenkins A, Bell M, Herzyk P, van der Weyden L, Adams DJ, Rust AG, Cameron E, Neil JC 2014 Insertional mutagenesis and deep profiling reveals gene hierarchies and a Myc/p53-dependent bottleneck in lymphomagenesis. PLoS Genet 10:e1004167. doi:10.1371/journal.pgen.1004167. 24586197 PMC3937229

[15] Uren AG, Kool J, Berns A, van Lohuizen M 2005 Retroviral insertional mutagenesis: past, present and future. Oncogene 24:7656–7672. doi:10.1038/sj.onc.1209043. 16299527

[16] Gilroy KL, Terry A, Naseer A, de Ridder J, Allahyar A, Wang W, Carpenter E, Mason A, Wong GK, Cameron ER, Kilbey A, Neil JC 2016 Gamma-retrovirus integration marks cell type-specific cancer genes: a novel profiling tool in cancer genomics. PLoS One 11:e0154070. doi:10.1371/journal.pone.0154070. 27097319 PMC4838236

[17] Jarrett O, Hardy WD Jr, Golder MC, Hay D 1978 The frequency of occurrence of feline leukaemia virus subgroups in cats. Int J Cancer 21:334–337. doi:10.1002/ijc.2910210314. 204584

[18] Sarma PS, Log T 1971 Viral interference in feline leukemia-sarcoma complex. Virology 44:352–358. doi:10.1016/0042-6822(71)90266-2. 18619364

[19] Mendoza R, Anderson MM, Overbaugh J 2006 A putative thiamine transport protein is a receptor for feline leukemia virus subgroup A. J Virol 80:3378–3385. doi:10.1128/JVI.80.7.3378-3385.2006. 16537605 PMC1440375

[20] Stewart MA, Warnock M, Wheeler A, Wilkie N, Mullins JI, Onions DE, Neil JC 1986 Nucleotide sequences of a feline leukemia virus subgroup A envelope gene and long terminal repeat and evidence for the recombinational origin of subgroup B viruses. J Virol 58:825–834. 3009890 10.1128/jvi.58.3.825-834.1986 PMC252989

[21] Boomer S, Eiden M, Burns CC, Overbaugh J 1997 Three distinct envelope domains, variably present in subgroup B feline leukemia virus recombinants, mediate Pit1 and Pit2 receptor recognition. J Virol 71:8116–8123. 9343161 10.1128/jvi.71.11.8116-8123.1997 PMC192267

[22] Rigby MA, Rojko JL, Stewart MA, Kociba GJ, Cheney CM, Rezanka LJ, Mathes LE, Hartke JR, Jarrett O, Neil JC 1992 Partial dissociation of subgroup C phenotype and in vivo behaviour in feline leukaemia viruses with chimeric envelope genes. J Gen Virol 73(Part 11):2839–2847. doi:10.1099/0022-1317-73-11-2839. 1331290

[23] Quigley JG, Burns CC, Anderson MM, Lynch ED, Sabo KM, Overbaugh J, Abkowitz JL 2000 Cloning of the cellular receptor for feline leukemia virus subgroup C (FeLV-C), a retrovirus that induces red cell aplasia. Blood 95:1093–1099. 10648427

[24] Tailor CS, Willett BJ, Kabat D 1999 A putative cell surface receptor for anemia-inducing feline leukemia virus subgroup C is a member of a transporter superfamily. J Virol 73:6500–6505. 10400745 10.1128/jvi.73.8.6500-6505.1999 PMC112732

[25] Onions D, Jarrett O, Testa N, Frassoni F, Toth S 1982 Selective effect of feline leukaemia virus on early erythroid precursors. Nature 296:156–158. doi:10.1038/296156a0. 6278316

[26] Anderson MM, Lauring AS, Burns CC, Overbaugh J 2000 Identification of a cellular cofactor required for infection by feline leukemia virus. Science 287:1828–1830. doi:10.1126/science.287.5459.1828. 10710311

[27] McDougall AS, Terry A, Tzavaras T, Cheney C, Rojko J, Neil JC 1994 Defective endogenous proviruses are expressed in feline lymphoid cells: evidence for a role in natural resistance to subgroup B feline leukemia viruses. J Virol 68:2151–2160. 8138999 10.1128/jvi.68.4.2151-2160.1994 PMC236690

[28] Simon V, Bloch N, Landau NR 2015 Intrinsic host restrictions to HIV-1 and mechanisms of viral escape. Nat Immunol 16:546–553. doi:10.1038/ni.3156. 25988886 PMC6908429

[29] Bogerd HP, Zhang F, Bieniasz PD, Cullen BR 2011 Human APOBEC3 proteins can inhibit xenotropic murine leukemia virus-related virus infectivity. Virology 410:234–239. doi:10.1016/j.virol.2010.11.011. 21131013 PMC3035163

[30] Stavrou S, Nitta T, Kotla S, Ha D, Nagashima K, Rein AR, Fan H, Ross SR 2013 Murine leukemia virus glycosylated Gag blocks apolipoprotein B editing complex 3 and cytosolic sensor access to the reverse transcription complex. Proc Natl Acad Sci U S A 110:9078–9083. doi:10.1073/pnas.1217399110. 23671100 PMC3670389

[31] Donahue PR, Hoover EA, Beltz GA, Riedel N, Hirsch VM, Overbaugh J, Mullins JI 1988 Strong sequence conservation among horizontally transmissible, minimally pathogenic feline leukemia viruses. J Virol 62:722–731. 2828667 10.1128/jvi.62.3.722-731.1988 PMC253625

[32] Geret CP, Cattori V, Meli ML, Riond B, Martinez F, Lopez G, Vargas A, Simon MA, Lopez-Bao JV, Hofmann-Lehmann R, Lutz H 2011 Feline leukemia virus outbreak in the critically endangered Iberian lynx (Lynx pardinus): high-throughput sequencing of envelope variable region A and experimental transmission. Arch Virol 156:839–854. doi:10.1007/s00705-011-0925-z. 21302124

[33] Sakaguchi S, Baba K, Ishikawa M, Yoshikawa R, Shojima T, Miyazawa T 2008 Focus assay on RD114 virus in QN10S cells. J Vet Med Sci 70:1383–1386. doi:10.1292/jvms.70.1383. 19122411

[34] Bailey P, Chang DK, Nones K, Johns AL, Patch AM, Gingras MC, Miller DK, Christ AN, Bruxner TJ, Quinn MC, Nourse C, Murtaugh LC, Harliwong I, Idrisoglu S, Manning S, Nourbakhsh E, Wani S, Fink L, Holmes O, Chin V, Anderson MJ, Kazakoff S, Leonard C, Newell F, Waddell N, Wood S, Xu Q, Wilson PJ, Cloonan N, Kassahn KS, Taylor D, Quek K, Robertson A, Pantano L, Mincarelli L, Sanchez LN, Evers L, Wu J, Pinese M, Cowley MJ, Jones MD, Colvin EK, Nagrial AM, Humphrey ES, Chantrill LA, Mawson A, Humphris J, Chou A, Pajic M, Scarlett CJ, Pinho AV, 2016 Genomic analyses identify molecular subtypes of pancreatic cancer. Nature 531:47–52. doi:10.1038/nature16965. 26909576

[35] Rojko JL, Fulton RM, Rezanka LJ, Williams LL, Copelan E, Cheney CM, Reichel GS, Neil JC, Mathes LE, Fisher TG, Cloyd MW 1992 Lymphocytotoxic strains of feline leukemia-virus induce apoptosis in feline T4-thymic lymphoma-cells. Lab Invest 66:418–426. 1349933

[36] Sheehy AM, Gaddis NC, Choi JD, Malim MH 2002 Isolation of a human gene that inhibits HIV-1 infection and is suppressed by the viral Vif protein. Nature 418:646–650. doi:10.1038/nature00939. 12167863

[37] Coffin JM, Haseltine WA 1977 Terminal redundancy and the origin of replication of Rous sarcoma virus RNA. Proc Natl Acad Sci U S A 74:1908–1912. doi:10.1073/pnas.74.5.1908. 68472 PMC431041

[38] Suspene R, Sommer P, Henry M, Ferris S, Guetard D, Pochet S, Chester A, Navaratnam N, Wain-Hobson S, Vartanian JP 2004 APOBEC3G is a single-stranded DNA cytidine deaminase and functions independently of HIV reverse transcriptase. Nucleic Acids Res 32:2421–2429. doi:10.1093/nar/gkh554. 15121899 PMC419444

[39] Rohn JL, Linenberger ML, Hoover EA, Overbaugh J 1994 Evolution of feline leukemia virus variant genomes with insertions, deletions, and defective envelope genes in infected cats with tumors. J Virol 68:2458–2467. 8139030 10.1128/jvi.68.4.2458-2467.1994 PMC236723

[40] Neil JC, Smart JE, Hayman MJ, Jarrett O 1980 Polypeptides of feline leukemia virus: a glycosylated gag-related protein is released into culture fluids. Virology 105:250–253. doi:10.1016/0042-6822(80)90173-7. 6251608

[41] Stavrou S, Crawford D, Blouch K, Browne EP, Kohli RM, Ross SR 2014 Different modes of retrovirus restriction by human APOBEC3A and APOBEC3G in vivo. PLoS Pathog 10:e1004145. doi:10.1371/journal.ppat.1004145. 24851906 PMC4031197

[42] Beale RC, Petersen-Mahrt SK, Watt IN, Harris RS, Rada C, Neuberger MS 2004 Comparison of the differential context-dependence of DNA deamination by APOBEC enzymes: correlation with mutation spectra in vivo. J Mol Biol 337:585–596. doi:10.1016/j.jmb.2004.01.046. 15019779

[43] Boomer S, Gasper P, Whalen LR, Overbaugh J 1994 Isolation of a novel subgroup B feline leukemia virus from a cat infected with FeLV-A. Virology 204:805–810. doi:10.1006/viro.1994.1597. 7941350

[44] Tsatsanis C, Fulton R, Nishigaki K, Tsujimoto H, Levy L, Terry A, Spandidos D, Onions D, Neil JC 1994 Genetic determinants of feline leukemia virus-induced lymphoid tumors: patterns of proviral insertion and gene rearrangement. J Virol 68:8296–8303. 7966623 10.1128/jvi.68.12.8296-8303.1994 PMC237298

[45] Bishop KN, Holmes RK, Malim MH 2006 Antiviral potency of APOBEC proteins does not correlate with cytidine deamination. J Virol 80:8450–8458. doi:10.1128/JVI.00839-06. 16912295 PMC1563846

[46] Schlesinger S, Goff SP 2015 Retroviral transcriptional regulation and embryonic stem cells: war and peace. Mol Cell Biol 35:770–777. doi:10.1128/MCB.01293-14. 25547290 PMC4323490

[47] Chaipan C, Dilley KA, Paprotka T, Delviks-Frankenberry KA, Venkatachari NJ, Hu WS, Pathak VK 2011 Severe restriction of xenotropic murine leukemia virus-related virus replication and spread in cultured human peripheral blood mononuclear cells. J Virol 85:4888–4897. doi:10.1128/JVI.00046-11. 21325415 PMC3126174

[48] Cruickshanks HA, McBryan T, Nelson DM, Vanderkraats ND, Shah PP, van Tuyn J, Singh Rai T, Brock C, Donahue G, Dunican DS, Drotar ME, Meehan RR, Edwards JR, Berger SL, Adams PD 2013 Senescent cells harbour features of the cancer epigenome. Nat Cell Biol 15:1495–1506. doi:10.1038/ncb2879. 24270890 PMC4106249

[49] Wu J, Pan TH, Xu S, Jia LT, Zhu LL, Mao JS, Zhu YL, Cai JT 2015 The virus-induced protein APOBEC3G inhibits anoikis by activation of Akt kinase in pancreatic cancer cells. Sci Rep 5:12230. doi:10.1038/srep12230. 26178819 PMC4503957

[50] Tubio JM, Li Y, Ju YS, Martincorena I, Cooke SL, Tojo M, Gundem G, Pipinikas CP, Zamora J, Raine K, Menzies A, Roman-Garcia P, Fullam A, Gerstung M, Shlien A, Tarpey PS, Papaemmanuil E, Knappskog S, Van Loo P, Ramakrishna M, Davies HR, Marshall J, Wedge DC, Teague JW, Butler AP, Nik-Zainal S, Alexandrov L, Behjati S, Yates LR, Bolli N, Mudie L, Hardy C, Martin S, McLaren S, O'Meara S, Anderson E, Maddison M, Gamble S, ICGC Breast Cancer Group, ICGC Bone Cancer Group, ICGC Prostate Cancer Group, Foster C, Warren AY, Whitaker H, Brewer D, Eeles R, Cooper C, Neal D, Lynch AG, Visakorpi T, 2014 Mobile DNA in cancer. Extensive transduction of nonrepetitive DNA mediated by L1 retrotransposition in cancer genomes. Science 345:1251343. doi:10.1126/science.1251343. 25082706 PMC4380235

[51] Onlamoon N, Das Gupta J, Sharma P, Rogers K, Suppiah S, Rhea J, Molinaro RJ, Gaughan C, Dong B, Klein EA, Qiu X, Devare S, Schochetman G, Hackett J Jr, Silverman RH, Villinger F 2011 Infection, viral dissemination, and antibody responses of rhesus macaques exposed to the human gammaretrovirus XMRV. J Virol 85:4547–4557. doi:10.1128/JVI.02411-10. 21325416 PMC3126245

[52] Mora M, Napolitano C, Ortega R, Poulin E, Pizarro-Lucero J 2015 Feline immunodeficiency virus and feline leukemia virus infection in free-ranging guignas (Leopardus guigna) and sympatric domestic cats in human perturbed landscapes on Chiloe Island, Chile. J Wildl Dis 51:199–208. doi:10.7589/2014-04-114. 25380363

[53] Zheng YH, Jeang KT, Tokunaga K 2012 Host restriction factors in retroviral infection: promises in virus-host interaction. Retrovirology 9:112. doi:10.1186/1742-4690-9-112. 23254112 PMC3549941

[54] Williams DK, Galvin TA, Gao Y, O'Neill C, Glasner D, Khan AS 2013 No evidence of xenotropic murine leukemia virus-related virus transmission by blood transfusion from infected rhesus macaques. J Virol 87:2278–2286. doi:10.1128/JVI.02326-12. 23236064 PMC3571456

[55] Mullins JI, Casey JW, Nicolson MO, Burck KB, Davidson N 1981 Sequence arrangement and biological activity of cloned feline leukemia virus proviruses from a virus-productive human cell line. J Virol 38:688–703. 6264136 10.1128/jvi.38.2.688-703.1981 PMC171199

[56] Tzavaras T, Stewart M, McDougall A, Fulton R, Testa N, Onions DE, Neil JC 1990 Molecular cloning and characterization of a defective recombinant feline leukaemia virus associated with myeloid leukaemia. J Gen Virol 71(Part 2):343–354. doi:10.1099/0022-1317-71-2-343. 2155287

[57] Overbaugh J, Riedel N, Hoover EA, Mullins JI 1988 Transduction of endogenous envelope genes by feline leukaemia virus in vitro. Nature 332:731–734. doi:10.1038/332731a0. 2895894

